# A role for neuromorphic processors in therapeutic nervous system stimulation

**DOI:** 10.3389/fnsys.2014.00187

**Published:** 2014-10-07

**Authors:** Corey M. Thibeault

**Affiliations:** Center for Neural and Emergent Systems, Information and System Sciences Laboratory, HRL Laboratories LLC.Malibu, CA, USA

**Keywords:** model-based control, neuromorphic hardware, deep brain stimulation, brain augmentation, spinal-cord stimulation

The motivations behind the development of many neuromorphic processors have been dominated by either the creation of better artificial intelligence, or novel non-von Neumann computing paradigms. A result of this impetus has been a number of low-power processors capable of simulating many different biological features of the nervous system. Power efficiency is crucial for deployed neuromorphic systems, but it also opens this technology up to other energy restricted applications. In this opinion, we suggest two such applications pertaining to therapeutic stimulation of the nervous system where closing the control loop could be assisted by advances in neuromorphic architectures: (1) deep brain stimulation (DBS) in the treatment of Parkinson's disease and (2) epidural spinal cord stimulation (ESS) for restoring voluntary motor functions. Though there are still questions that must be addressed before this would be feasible, but we are suggesting that the technological barriers—in both the algorithms and hardware—can be overcome with directed funding and research.

Neuromorphic processor research is centered around the creation of brain-like intelligence through power-efficient circuits that borrow elements directly from biology (Mead, 1989). The applications for these projects range from brain-scale simulations (Gao et al., 2012; Benjamin et al., 2014) and *in silica* experimentation (Schemmel et al., 2010; Furber et al., 2012), to brain-like computing and learning (Merolla et al., 2011; Srinivasa and Cruz-Albrecht, 2012; Cruz-Albrecht et al., 2013; Rahimi Azghadi et al., 2014; Schmuker et al., 2014). These projects promise unrivaled access to large-scale models of the brain as well as insight into the unique non-von Neumann computation that biological systems appear to achieve.

Regardless of the motivation, the tangible result of these efforts has been an accumulation of low-power circuits capable of emulating various elements of the nervous system. Although these are essential for embodying robotic systems and augmenting current super-computing paradigms, they also have the potential to assist in nervous system stimulation control. This application is outside the scope of the currently funded neuromorphic hardware projects, but with new insights and technological advances, it is one that will be particularly beneficial.

## 1. Model based control

**Figure 1:**
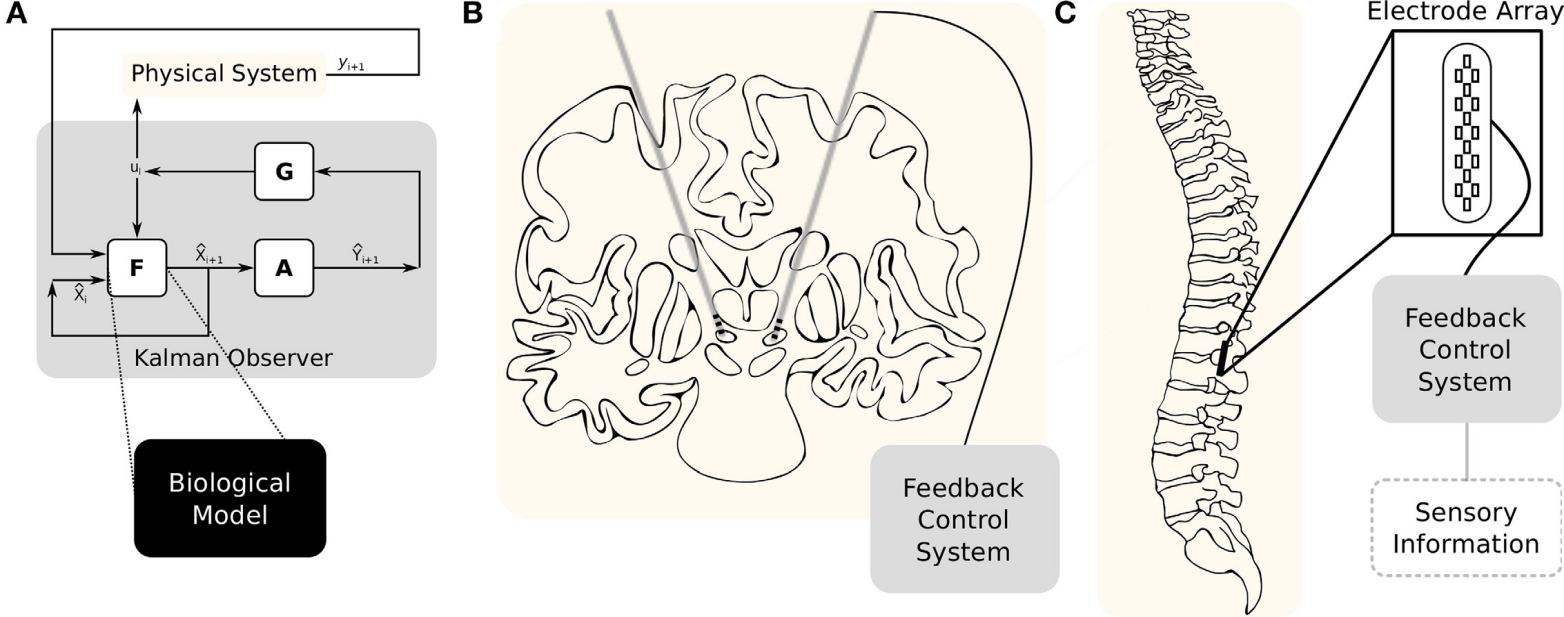
**Example therapeutic applications of model based control. (A)** The system dynamics are described by a model, *F*, and the observations are described by a function, *A*. In most systems those observations are going to be noisy, so a covariance matrix, *R*, will account for that. After one step of *F*, using the resulting sigma points will provide ${\tilde{X}}_{i}$ = *F*(*X*_*i*_). A new set of observations can then be found, *Ỹ_i_* = *A*(*X_i_*). The means over these two matrices are the *a priori* state and measurement estimates. The *a posteriori* state estimate, $\hat{x}$, is now dependent on the state estimate, $\tilde{x}$, the measurement estimate, ỹ, the actual measurement, *y*, and the Kalman gain matrix, *G*. **(B)** Diagram of deep brain stimulation in the treatment of Parkinson's disease. Adapted from Thibeault and Srinivasa (2013). **(C)** Example epidural spinal cord stimulation for restoring voluntary motor functions.

In our current capacity to monitor neural circuits, most system variables are unobservable. One strategy for estimating these unknown system variables and parameters is by employing an Unscented Kalman Filter (UKF) to combine the observable and unobservable states. The UKF employs a set of known dynamical equations and observation functions with the measurable data to update an approximation of the state and its uncertainty. At each update, sigma points—system states that are consistent with the current state uncertainty—are selected and used to integrate the system. These are combined with estimated mean state values and the approximate uncertainty. A gain matrix then updates the new most likely state of the system. The schematic for this organization is illustrated in Figure 1A. Applying this kind of feedback control to biological systems was initially demonstrated by Voss et al. (2004) but has since been demonstrated on a number of control and estimation problems (Abarbanel et al., 2008; Li et al., 2009; Ullah and Schiff, 2009, 2010; Schiff, 2010; ODoherty et al., 2011; Aprasoff and Donchin, 2012; Schiff, 2012; Liu et al., 2014).

By using a model of the area under stimulation, both the activity and state of that area can be approximated—something that is not directly measurable. The model, constructed from the current understanding of the anatomy, can then be used to find an optimal set of stimulation parameters. In addition, the model output can be used as the feedback into a control system that can not only dynamically tune the stimulation parameters but also adapt to the physiological circuit remodeling—providing the highest possible therapeutic benefit. Embedding these models in low-power neuromorphic hardware would facilitate a transition into implantable devices.

A discussion of control inherently implies observability of the system. However, observability alone is useful to current nervous system stimulation strategies. Observing the unknown—or unreachable—states of the physical system, would provide a way to automatically tune the stimulation parameters—assisting clinicians in finding the optimal set points in open-loop control. Finally, in addition to the UKF there are other model-based control schemes that could be employed here.

## 2. Deep brain stimulation in the treatment of parkinson's disease

The application of DBS to patients with pharmacoresistant Parkinson's disease can be traced back to the early 1980's (Montgomery Jr, 2012). In DBS, dual electrodes are implanted bilaterally into the nuclei of the basal ganglia (see Figure 1B)—the current target is the subthalamic nucleus. Constant electrical pulses are then injected into the electrodes. After implantation, clinicians will experiment with frequency, amplitude, and duration of those electrical pulses to find a configuration with the highest benefit. Finding that point however, is an inexact science and periodic adjustments to compensate for neural plasticity are required. Although there is a proven clinical benefit to DBS, there is no clear explanation for its mechanism of action.

Although the open-loop configuration of DBS has proven capable, closed-loop control of DBS has been shown to be a more effective treatment in both theoretical (Santaniello et al., 2011), and physiological experiments (Rosin et al., 2011). For example, in Rosin et al. (2011) a simple feedback loop was created where the activation of the DBS pulse was triggered by spiking in a reference structure—either the internal segment of the globus pallidus or primary motor cortex. The control paradigm demonstrated a larger reduction in pallidal oscillations and akinesia compared to open-loop DBS. The resulting system—although brilliantly designed—is an incredibly simple solution and one that exemplifies the therapeutic advances that can be made with adaptive feedback control systems.

The class of model-based control of DBS suggested here has already been demonstrated in simulation space by Schiff (2010) using the simple neuron implementation of Rubin and Terman (2004). Although the mathematical model used in that study was computationally cheaper than the alternative, it is still difficult to simulate in a low-power microprocessor. Aspects of the original Rubin and Terman (2004) results were implemented using a more hardware friendly model in Thibeault and Srinivasa (2013), however, the required level of abstraction in a control paradigm is still unclear. Despite unanswered questions, these studies are encouraging and demonstrate the feasibility of the strategy.

## 3. Epidural spinal cord stimulation

The recent discoveries in the use of epidural spinal cord stimulation—diagrammed in Figure 1C—on patients with motor complete paraplegia has revealed a therapeutic pathway toward restoring voluntary motor function (Harkema et al., 2011). However, the mechanisms behind this benefit as well as the supporting technology is still immature. The current state-of-the-art involves randomly tuning the stimulation parameters manually until a physiological improvement is observed—these parameters include both the duration and amplitude of the stimulus as well as anode/cathode pairings. There have been efforts to apply Bayesian optimization approaches to automating the parameter search but these did not directly account for the relevant biological structures (Desautels, 2014).

Additionally, it has been suggested that the therapeutic restoration of motor control is mechanistically dependent on the remodeling of the remaining spinal circuits (van den Brand et al., 2012). Having a control strategy as well as a model that are adaptive to the plastic changes within the spinal circuits would require less manual parameter adjustments over the life of the implant.

As a clinical treatment, ESS is still underdeveloped. However, it is one that could benefit from a model-based control strategy—either as an observer system for parametric optimization or as a complete closed-loop solution. Although the fidelity of the spinal cord model and the source of sensory feedback have not been fully explored, in many ways this appears to be a more tractable problem compared to DBS—it may also prove to be an ideal alternative as well (Fuentes et al., 2009). The accessibility of the spinal cord as well as the simplicity of the microcircuit may make closing the loop on ESS more feasible. However, if more finely tuned control of the individual muscles is required, the complexity of the problem could quickly out pace that of DBS.

## 4. Conclusion

Despite the technological and theoretical advances outlined here, there are still obstacles to overcome. Where and what to measure when closing the loop for both DBS and ESS is not entirely clear. The stability of the hardware measuring those signals is also a concern. Furthermore, as mentioned above, the appropriate level of biological fidelity required in the model has not been fully resolved. The proposed use of neuromorphic hardware implies that the model for the control system actually requires high-fidelity. In DBS treatment of Parkinson's disease this appears to be the case. However, for spinal-cord stimulation, it may not be required. Regardless, closed-loop strategies are clearly more effective and the theoretical and technological barriers are low enough that a concerted effort should be made to advance this concept toward clinical treatments.

Finally, model-based control strategies will not only improve the therapeutic benefit but the power consumption as well. Rather than blindly applying stimulation, pulses can be applied only as needed. Utilizing neuromorphic hardware will add to that power savings by both reducing the computational burden and providing the necessary biological detail for model-based control.

### Conflict of interest statement

The author declares that the research was conducted in the absence of any commercial or financial relationships that could be construed as a potential conflict of interest.
